# Level of disability and associated factors among stroke survivors in Ethiopia: a multicenter cross-sectional study

**DOI:** 10.1186/s12889-025-26147-w

**Published:** 2026-01-05

**Authors:** Getachew Azeze Eriku, Chalachew Mersha, Mihret Dejen Takele, Destaw Marie Merawie, Ermias Solomon Yalew, Solomon Gedlu Nigatu, Alemakef Wagnew Melesse, Tesfa Kassa

**Affiliations:** 1 Department of Physiotherapy, School of Medicine, College of Medicine and Health Sciences, University of Gondar, P.O. Box: 196, Gondar, Ethiopia; 2 Department of Physiotherapy, University of Gondar Comprehensive Specialized Hospital, University of Gondar, Gondar, Ethiopia; 3 Department of Epidemiology and Biostatistics, Institute of Public Health, College of Medicine and Health Sciences, University of Gondar, Gondar, Ethiopia

**Keywords:** Stroke, Stroke survivors, Level of disability, Post-stroke disability, Severity of disability, Ethiopia

## Abstract

**Background:**

Stroke is a leading cause of long-term disability worldwide, significantly affecting the physical, cognitive and emotional well-being of stroke survivors. Post-stroke disability often leads to limitations in daily activities, reduced social participation, and decreased quality of life. These result from complex interactions between an individual’s health condition and their environments. Despite this substantial burden, limited data exist on the severity of disability among stroke survivors in Ethiopia. Therefore, this study aims to assess the level of disability among stroke survivors in Ethiopia.

**Methods and materials:**

A multicenter institutional-based cross-sectional study was conducted from July 1 to August 31, 2024 in five public hospitals in Amhara Regional State. A total of 292 participants were selected using systematic random sampling. Disability was assessed using the 12-item World Health Organization Disability Assessment Schedule 2.0 (WHODAS2.0). Ordinal logistic regression was performed to identify factors associated with the level of disability.

**Results:**

Of the 292 stroke survivors, 17.5% of participants (95% CI: 13.0- 21.9) had mild, 43.8% (95% CI: 38- 49.7) moderate, and 38.7% (95% CI: 32.9–44.5) severe disability. Factors significantly associated with higher disability included age above 50 years (AOR = 2.87; 95% CI: 1.71–4.79), rural residence (AOR = 3.49; 95% CI: 1.65–7.40), stroke onset less than six months (AOR = 2.81; 95% CI: 1.69–3.45), presence of comorbidities (AOR = 1.94; 95% CI: 1.08–3.45), use of assistive devices (AOR = 2.69; 95% CI: 1.61–4.52), and longer hospitalization (AOR = 2.07; 95% CI: 1.22–3.52).

**Conclusions and recommendations:**

A high burden of moderate to severe disability was reported among stroke survivors. Older adults, individuals residing in rural areas, those in the early stage of recovery, those with comorbid conditions, those rely on assistive devices for mobility, and those with prolonged hospitalization require particular attention. These findings highlight the need for disability-inclusive public health strategies, expanded rehabilitation services, and equitable access to care, particularly in rural and resource-limited settings. Future studies with larger sample sizes, longitudinal designs, and community-based approaches are recommended to better understand disability trajectories and inform targeted interventions.

## Background

Stroke is a major neurological disorder caused by acute vascular injury of the central nervous system, including cerebral infarction, intracerebral hemorrhage, and subarachnoid hemorrhage [1]. It is a leading cause of death and long-term disability, imposing significant physical, cognitive and emotional burdens on stroke survivors globally [2, 3]. According to the 2021 Global, Regional, and National Burden of Stroke Report, stroke is the third leading cause of death, after ischemic heart disease and COVID-19, and the fourth leading cause of disability-adjusted life years (DALYs). Over 80% of incident strokes, more than 75% of prevalent cases, approximately 85% stroke-related deaths, and nearly 90% of stroke-related DALYs occur in low-and middle-income countries (LMICs), where access to healthcare and rehabilitation services are often limited [4]. The rising burden of stroke is largely attributed to population aging and increased exposure to modifiable risk factors, including hypertension, diabetes mellitus, obesity, air pollution, and behavioral factors [5].

Disability is increasingly recognized as a major public health and development issue, particularly in LMICs such as Ethiopia [6, 7]. Beyond its clinical consequences, disability affects population health, social participation, economic productivity, and overall national development [6, 8]. People with disabilities often experience poor health outcomes, limited access to health and rehabilitation services, reduced employment opportunities, and increased risk of poverty, largely due to structural and social barriers [9, 10]. In response, disability has been integrated into global development agendas, including the Sustainable Development Goals, which emphasize equity, inclusion, and universal access to health services [11]. In countries like Ethiopia, where rehabilitation services and assistive technologies are limited, disability from chronic conditions such as stroke poses a substantial public health challenge, underscoring the need for evidence to guide disability-inclusive health policies and service planning [12–14].

Globally, approximately 90% of stroke survivors experience some form of disability [15]. Stroke can impair bodily structures and functions across physical, cognitive, and emotional domains, significantly limiting daily activities and reducing their overall quality of life. The severity of disability is a critical determinant of recovery and the ability to return to work or social roles. Functional outcomes vary according to subtype, initial severity, and access to timely and effective rehabilitation services [16]. In Ethiopia, higher levels of disability have been associated with lower health-related quality of life (HRQOL) [12, 13]. Caregivers of people with disabilities frequently experience psychological distress due to the caregiving burden [17, 18].

Evidence indicates that the functional outcomes differ notably between ischemic and hemorrhagic stroke [19], with hemorrhagic stroke often resulting in greater caregiver burden [20]. A longitudinal study found that a significant proportion of stroke survivors continue to experience moderate to severe disability even a decade after the event [21]. In addition, many stroke survivors face depression and anxiety [22], chronic pain [23], collectively affecting overall HRQOL [24].

Although understanding the multifaceted nature of post-stroke disability and its contributing factors is essential for early interventions, rehabilitation planning, evidence on the extent and severity of disability among stroke survivors in Ethiopia remains limited. Therefore, this study aims to assess the level of disability and its associated factors among stroke survivors attending public hospitals in Ethiopia, where access to and quality of rehabilitation services are limited.

## Methods and materials

### Study design, setting, and period

A multicenter cross-sectional study was conducted from July 1 to August 31, 2024, among stroke survivors attending public hospitals in Amhara Regional State, Ethiopia. According to the Amhara National Regional Health Bureau, the region comprises 81 hospitals, 858 health centers, and 3,560 health posts. Of these hospitals, eight are referral and comprehensive specialized hospitals that provide both medical follow-up and physiotherapy treatment for stroke survivors and were eligible for inclusion in the study.

From these eight eligible hospitals, the University of Gondar Comprehensive Specialized Hospital (UOGCSH), Felege-Hiwot Comprehensive Specialized Hospital (FHCSH), Tibebe Ghion Comprehensive Specialized Hospital (TGCSH), Debre-Markos Comprehensive Specialized Hospital (DMCSH), and Debre-Tabor Comprehensive Specialized Hospitals (DTCSH) were randomly selected for this study.

UOGCSH is located in Gondar city, Amhara region, approximately 738 km northwest of Addis Ababa. TGCSH and FHCSH are located in Bahir Dar, the capital of the Amhara National Regional State, about 565 km from Addis Ababa. DMCSH is found in Debre Markos City, roughly 300 km from Addis Ababa and 250 km from Bahir Dar. DTCSH is located in Debre Tabor city, approximately 100 km from Bahir Dar and about 650 km from Addis Ababa. Currently, the comprehensive specialized hospitals in the Amhara region serve a catchment population of more than 20 million people, including those from neighboring regions.

### Source and study population

#### Source population

The source population comprised adult stroke survivors attending medical and physiotherapy outpatient clinics at public hospitals in the Amhara Regional State. The study population included adult stroke survivors attending medical and physiotherapy outpatient follow-up clinics at UOGCSH, TGCSH, FHCSH, DTCSH, and DMCSH during the data collection period.

#### Inclusion and exclusion criteria

All stroke survivors aged ≥ 18 years who were at least two months post-stroke onset were included in the study. Stroke survivors diagnosed with dementia, those with severe cognitive impairments or aphasia limiting their communication, and individuals diagnosed with neurological conditions other than stroke were excluded from the study. These conditions were identified based on medical chart documentation and clinical reports.

#### Sample size determination

Based on one-month outpatient attendance record from each hospital, the average number of stroke survivors attending medical and physiotherapy outpatient departments was 120 at UOGCSH, 105 at TGCSH, 103 at FHCSH, 80 DTCSH, and 95 at DMCSH. Accordingly, during the two-month data collection period, the estimated number of stroke survivors was 240, 210, 206, 160, and 190 for UOGCSH, TGCSH, FHCSH, DTCSH, and DMCSH, respectively, giving a total estimate of 1,006 stroke survivors.

The required sample size was calculated using a single population proportion formula, assuming a prevalence of 50% (due to the absence of prior studies in Ethiopia), a 95% confidence level (95% CI), and a 5% margin of error. This yielded an initial sample size of 385. Since the population was fewer than 10,000, a finite population correction was applied, resulting in an adjusted sample size of 278. After accounting for a 10% non-response rate, the final sample size was determined to be 306.

The final sample size was calculated as shown below: $\:\mathrm{n}=\frac{{{(\mathrm{Z}\mathrm{a}}_{/2})}^{2}\mathrm{*}\mathrm{P}\left(1-\mathrm{P}\right)}{{\mathrm{d}}^{2}}=\frac{{\left(1.96\right)}^{2}\mathrm{*}\:0.5\left(1-0.5\right)}{{\left(0.05\right)}^{2}}=384.16\sim385$

Where n = sample size, P = prevalence and d = margin of error.

Since the total population was less than 10,000, the finite population correction formula was applied.

$$\:{\mathrm{n}}^{{\prime\:}}=\frac{\mathrm{n}}{1+\frac{1}{\mathrm{N}}}=\frac{385}{1+\frac{385}{1006}}=278$$

Considering a 10% non-response rate, the final sample size was adjusted to: 278 + 28 (10% of 278) = 306.

### Sample technique and procedure

Study participants were proportionally allocated to each selected hospital based on the estimated number of stroke survivor attending outpatient clinics during the study period. Over the two-month data collection period, a total of 1006 stroke survivors visited the selected hospitals. Specifically, 240, 210, 206, 160, and 190 stroke survivors visited the medical and physiotherapy outpatient departments of UOGCSH, TGCSH, FHCSH, DTCSH, and DMCSH, respectively.

Systematic random sampling was employed to select participants. The sampling interval (K) was calculated by dividing the total estimated population by the final sample size (1006/306), which is approximately 3. Accordingly, every 3rd eligible stroke survivor was selected. The first participant was chosen randomly using a lottery method. Based on proportional allocation, 69 participants were selected from UOGCSH, 65 from TGCSH, 64 from FHCSH, 50 from DTCSH, and 58 from DMCSH.

### Data collection tool

The data collection tool was developed based on a review of relevant literature [13, 25–28] and consisted of three components: sociodemographic characteristics, stroke-related information, and level of disability. Data were collected using face-to-face interviews and medical chart reviews. Sociodemographic information was obtained through interviews, while clinical information, including the type of stroke, length of hospitalization, and the presence of comorbidities, was extracted from medical records.

Disability was measured using the 12-item WHODAS 2.0, which has been culturally adapted, validated, and tested for reliability in Ethiopia. Previous Ethiopian studies have demonstrated good psychometric properties among Amharic-speaking populations, including acceptable internal consistency, construct validity and test-retest reliability [29, 30]. In this study, the validated Amharic version was used without modification.

The WHODAS 2.0 assesses disability across six domains: cognition, mobility, self-care, getting along, life activities, and participation with two items per domain. Disability experienced during the preceding 30 days was rated on a five-point Likert-type scale, ranging from 1 (no difficulty) to 5 (extremely difficult) [31]. Item scores were summed and transformed into a total score ranging from 0 to 100, where 0 indicates no disability and 100 indicates extremely severe disability.

Based on established cut-off points in a similar study [32], level of disability was categorized as no disability (0–4), mild (5–24), moderate (25–49), severe (50–95), and extremely severe disability (96–100). In this study, no participants were classified as having no disability, and only a few fell into the extremely severe disability category. Therefore, the five original categories were collapsed into three levels: mild, moderate, and severe disability, for subsequent analysis.

### Data quality assurance

The questionnaire was initially prepared in English, translated into Amharic, and finally back-translated into English to ensure consistency. Data were collected by trained physiotherapists under daily supervision. Data collectors and supervisors received one-day training prior to the actual data collection period. A pre-test was conducted at Ayera General Hospital in Gondar city on 5% of the total sample size in early June 2024. Based on the pre-test findings, necessary amendments were made, including rewording ambiguous questions, simplifying technical terms, and correcting grammar and punctuation errors.

The principal investigator regularly checked the collected data for completeness, accuracy, and clarity. Data consistency and completeness were monitored throughout the data collection, entry, and analysis.

### Data analysis

Data were checked, cleaned, and entered into EPI-data version 4.4.3.1 and analyzed using STATA version 17. Descriptive statistics (mean, standard deviation, frequencies, and percentages) were used to summarize participant characteristics. Normality of continuous variables was assessed using the Shapiro-Wilk test. Multicollinearity was evaluated using the variance inflation factor (VIF) with a cutoff point of VIF < 10. Model goodness of fit was assessed using the Hosmer-Lemeshow test (*p*-value = 0.49).

Ordinal logistic regression was applied to identify factors associated with the level of disability. The proportional odds assumption (parallel lines) was tested and satisfied before fitting the final ordinal logistic regression model. Variables with a *p*-value < 0.25 in bivariable analysis were included in the multivariable analysis. Adjusted ratios (AOR) with 95% CI were reported, and a *p*-value < 0.05 was considered statistically significant.

## Results

### Sociodemographic characteristics of stroke survivors

A total of 292 stroke survivors participated in this study, yielding a response rate of 95.4%. More than half the participants were male and aged 50 years or older, and nearly one-third resided in urban areas. Nearly two-thirds of the study participants were married and had a low household income of less than ETB 5000. See Table 1.

**Table 1:** Sociodemographic characteristics of stroke survivors in Amhara regional state public Hospitals, Ethiopia (*N* = 292)

Variables	Categories	*N* (%)
Sex	Male	164(56.2)
	Female	128(43.8)
Age	50 and below	121(41.4)
	Above 50	171(58.6)
Residence	Urban	192(65.8)
	Rural	100(34.2)
Marital status	Married	202 (69.2
	Not married	90 (30.8)
Educational status	No formal education	87 (29.8)
	Primary	72 (24.7)
	Secondary	51 (17.8)
	Diploma and above	81(27.7)
Employment	Government employed	80 (27.4)
	Housewife	71 (24.3)
	Non-governmental	109 (25.7)
	Farmer	56 (19.2)
	Retired	30 (10.3)
Family monthly income	< 5000	205 (70.2)
	5000 and above	87 (29.8)

### Stroke-related characteristics of stroke survivors

More than half of the stroke survivors were diagnosed with ischemic stroke and had right-sided lesions. The majority had experienced their stroke more than six months prior to data collection and had one or more comorbidities. Two-thirds of the participants had been hospitalized for longer than one week. Additionally, over half of the stroke survivors relied on assistive devices for mobility. See Table 2.

**Table 2:** The stroke-related characteristics of stroke survivors in Amhara regional state public hospitals, Ethiopia

Variables	Category	*N* (%)
Type of stroke	Ischemic	174 (59.6)
	Hemorrhagic	118(40.4)
Side of lesion	Left	138(47.3)
	Right	154(52.7)
Duration of stroke	< 6 months	113 (38.7)
	> 6 months	179 (61.3)
Length of hospitalization	One week and less	96(32.9)
	More than one week	196(67.1)
Comorbidities	Yes	214(73.3)
	No	72(26.7)
Assistive devices	Yes	156(53.4)
	No	136(46.6)

### Level of disability among stroke survivors

**Fig. 1:**
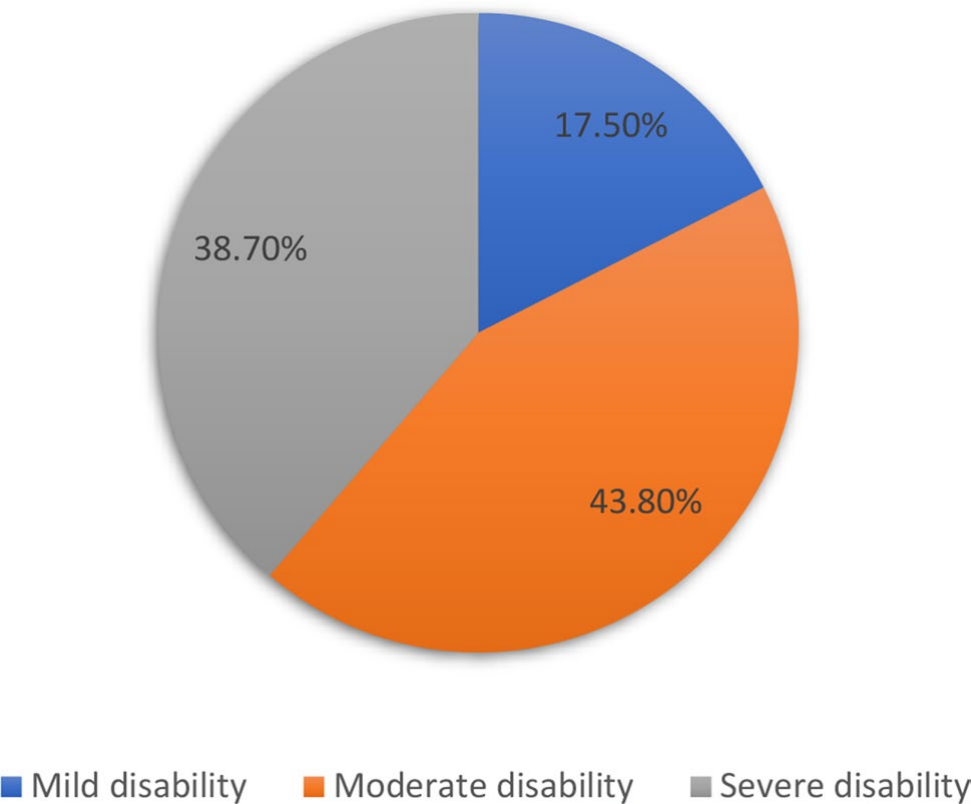
Level of disability among stroke survivors in Amhara Regional state public hospitals, Ethiopia

Our study found that 17.5% (51) (95% CI: 13.0- 21.9) of stroke survivors were categorized as having mild disability, 43.8% (128) (95% CI: 38- 49.7) moderate disability and 38.7% (113) (95% CI: 32.9–44.5) had severe disability. Overall, more than two-thirds of participants experienced moderate to severe disability, as measured by the 12-item WHODAS 2.0. See Fig. 1.

### Factors associated with the level of disability among stroke survivors

Several factors were significantly associated with higher levels of disability among stroke survivors, including older age, rural residence, shorter duration since stroke onset, presence of comorbidities, use of assistive devices for mobility and longer hospitalization.

Stroke survivors aged 50 years and above had 2.87 times higher odds of experiencing severe disability compared to those under 50 (AOR = 2.87; 95% CI: 1.71–4.79, *p* < 0.001). Those residing in rural areas were 3.49 times more likely to experience severe disability than their urban counterparts (AOR = 3.49; 95% CI: 1.65–7.40, *p* = 0.001). Survivors with a stroke onset of less than six months had nearly three times higher odds of severe disability compared to those with a longer duration since onset (AOR = 2.81; 95% CI: 1.69–3.45, *p* < 0.001 ).

The presence of comorbidities was also significantly associated with increased disability; stroke survivors with one or more comorbidities had nearly 1.94 times the odds of severe disability compared to those without comorbidities (AOR = 1.94;95% CI: 1.08–3.45, *p* = 0.026). Similarly, those using assistive devices for mobility had 2.69 times higher odds of severe disability than those who did not (AOR = 2.69; 95% CI: 1.61–4.52, *p* < 0.001).

Lastly, hospitalization for more than one week was associated with 2.07 times higher odds of severe disability compared to shorter stays (AOR = 2.07; 95% CI: 1.22–3.52, *p* = 0.007). See Table 3.

**Table 3:** The factors associated with level of disability among stroke survivors public hospitals in Amhara regional State, Ethiopia (*N* = 292)

Variables	Category	Level of disability			AOR(95%CI)	P> (z)
		Mild	Moderate	Severe		
Age	Less than 50	35(28.9)	61(50.4)	25(20.7)	1	
	Above 50	16(9.4)	67(39.2)	88(51.5)	2.87(1.71,4.79)	<0.001
Sex	Male	38(23.2)	66(40.2)	60(36.6)		
	Female	13(10.2)	62(48.4)	53(41.1)	1.26(0.75,2.09)	0.37
Marital status	Married	41(20.3)	84(41.6)	77(38.1)	1	
	Unmarried	10(11.1)	44(48.9)	36(40)	1.17(0.68,2.02)	0.55
Residency	Urban	46(24)	93(48.4)	53(27.6)	1	
	Rural	5(5)	35(35)	60 (60)	3.49(1.65,7.40)	< 0.001
Education	Unable to read and write	9(10.3)	26(29.9)	52(59.8)	1.75(0.70,4.34)	0.23
	Primary	8(11.1)	35(48.6)	29(40.3)	1.31(0.62,2.75)	0.47
	Secondary	5(9.6(	33(63.5)	14(26.9)	1.52(0.74,3.10)	0.25
	Diploma and above	29(35.8)	34(42)	18(22.2)	1	
Income	5000 and less	28(13.7)	88(42.9)	89(43.3)	0.81(0.46,1.41)	0.45
	Above 5000	23(26.4)	40(46.0)	24(27.6)	1	
Duration since stroke onset	Less than 6 months	13(11.5)	43(38.1)	57(50.4)	2.81(1.69,4.66)	< 0.001
	Duration more than 6 months	38(21.2)	85(47.5)	56(31.3)	1	
Comorbidities	Yes	25(11.7)	99(46.3)	90(42.1)	1.94(1.08,3.45)	0.026
	No	26(33.3)	29(37.2)	23(29.5)		
Assistive device	Yes	16(10.3)	72(46.2)	68(43.6)	2.69(1.61,4.52)	< 0.001
	No	35(25.7)	56(41.2)	45(33.1)	1	
Length of hospitalization	Less than one week	34(35.4)	32(33.3)	30(31.3)	1	
	More than one week	17(8.7)	96(49)	83(42.3)	2.07(1.22,3.52)	0.007

## Discussion

This study assessed the level of post-stroke disability and its associated factors among stroke survivors’ public hospitals of the Amhara region, Ethiopia. The findings showed that 17.5% of stroke survivors had mild disability, 43.8% had moderate disability, and 38.7% had severe disability. Overall, more than four-fifths (82.5%) of participants experienced moderate to severe disability. This finding is consistent with a study from Bangladesh, in which 80.8% of participants reported moderate to severe disability [33]. This similarity may be attributed to the hospital-based nature of both studies and the high proportion of participants with comorbid conditions, with more than three-fourths reporting at least one comorbidity.

The prevalence reported in this study is higher than that reported in a study from Nigeria, where 47% of stroke survivors had disability [27]. The discrepancy may reflect difference in operational definitions and analytical methods. The Nigerian study used a binary cutoff score of 45 and binary logistic regression, whereas our study used ordinal logistic regression and categorized disability into multiple levels. Our finding is also higher than that of a follow-up study in China, which reported an initial disability prevalence of 63.8%, declining to 47% after a three years [25]. This difference may reflect disparities in healthcare infrastructure and rehabilitation services. In China, regular follow-up and access to rehabilitation services likely contributed to improved functional outcomes, whereas in Ethiopia, access to such services remains limited. Additionally, differences in disability assessment tools may have contributed. The present study used the 12-item WHODAS 2.0, which assesses multiple functional domains including cognition, mobility, self-care, getting along with others, life activities and participation [34] and may identify mild to moderate disabilities that are overlooked by Barthel index, which focuses primarily on basic activities of daily living. As a result WHODAS 2.0 may provide a more comprehensive assessment of disability, contributing to the higher reported disability prevalence.

Several factors were significantly associated with post-stroke disability, including older age, rural residence, shorter duration since stroke onset, presence of comorbidities, use of assistive devices for mobility and longer hospitalization.

Stroke survivors over 50 years had significantly higher odds of severe disability, consistent with previous Ethiopian studies and global evidence that identifying advanced age as a strong predictor of post-stroke functional limitations [35–37]. A study conducted in Ghana similarly reported a significant association between age and level of disability, with most individuals experiencing moderate to severe disability falling within the age range of 48 to 63 years [26]. Comparable findings have also been consistently reported in studies in china [25, 28, 38]. The association between advanced age and severe disability may explained by age-related physiological changes and diminished neuroplasticity, which limit the brain’s ability to reorganize and functional recovery following stroke [39–42].

Stroke survivors from rural areas were more likely to experience severe disability than their urban counterparts. This may be explained by inequities in healthcare access between rural and urban settings. Rural residents often face geographic barriers, limited availability of healthcare facilities, inadequate rehabilitation services, and restricted access to assistive devices, all of which may contribute to poorer functional outcomes. These findings are supported by a study from South Africa that linked rural residence to increased disability severity due to limited access to healthcare, education, and employment opportunities, and basic services [43]. Similarly, a study from Colombia reported that rural stroke survivors experienced more severe strokes and poorer functional outcomes at discharge and three months post-stroke compared to urban residents [44].

The level of disability was also significantly associated with the time since stroke onset. Survivors assessed within six months of stroke onset were nearly three times more likely to report severe disability than those assessed later. This may be explained by the early stage of recovery, which is typically characterized by limited functional improvement, ongoing neurological impairments, and reduced access to or engagement with rehabilitation services. As recovery progresses, functional improvements generally occur due to neuroplasticity and the cumulative effects of rehabilitation.

This finding is consistent with a study from Thailand, which reported an increase in independence from 5.5% at discharge to 25.5% at six and twelve months post-stroke [45]. Another study noted that the most rapid neurological and functional recovery tends to occur within the first three months, during which patients often experience substantial impairments, resulting in higher observed levels of disability [46]. Continued rehabilitation, neuroplastic changes, and natural healing processes contribute to gradual functional improvement overtime, thereby reducing the severity of disability among those assessed after six months [47, 48].

Comorbid conditions significantly increased the odds of severe disability. Stroke survivors with one or more comorbidities were nearly twice as likely to report severe disability compared to as those without comorbid conditions. A possible explanation is that comorbidities impose an additional physiological burden by impairing cardiovascular and metabolic functions, reducing endurance and complicating the recovery process. Additionally, premorbid functional decline may further limit active participation in rehabilitation. These findings are consistent with studies from Sweden [49] and the United Kingdom [50], which identified comorbidities and premorbid disability as a strong predictors of poor functional outcomes among stroke survivors.

In this study, the use of assistive devices for mobility was associated with higher odds of severe disability. Those who relied on mobility aids were 2.7 times more likely to report severe disability than those who did not. This association likely reflects the severity of underlying impairments, such as motor deficits, balance dysfunction, or cognitive impairments. In contexts like Ethiopia, limited training on proper device use and a lack of assistive technology services may contribute to increased dependency and further functional restrictions. Studies have shown that stroke survivors who require mobility aids tend to have lower functional independence scores [51], poorer balance and higher fall risk [52]. Other studies have found a strong correlation between assistive device use and dependence in daily activities [53].

Prolonged hospitalization was another key factor associated with severe disability. Stroke survivors hospitalized for more than one week had significantly higher odds of severe disability compared to those with shorter stays. This may reflect greater initial severity, the occurrence of secondary complications, and delayed access to early rehabilitation services. Previous studies have shown that prolonged hospitalization is associated with poor functional outcomes at discharge [54]. This association may also be mediated by immobility as prolonged bed rest leads to muscle atrophy, reduced cardiovascular fitness, and decreased endurance, all features of hospital-associated deconditioning. Evidence from a systematic review and meta-analysis indicates that early rehabilitation and mobilization can prevent some of these physiological consequences and improve recovery outcomes, underscoring the importance of timely rehabilitation during the acute phase of stroke care [55]. Additionally, a study from the U.S found that hospitalization-associated disability is common but often under recognized in older adults, particularly with following prolonged bed rest [56].

In resource-limited settings like Ethiopia, the problem may be exacerbated by inadequate acute care, delayed initiation of rehabilitation services, and poor post-discharge follow-up, all of which increase the risk of persistent disability. From a public health perspective, the high burden of moderate to severe disability observed in this study highlights substantial gaps in rehabilitation coverage and disability-inclusive health services in Ethiopia. Limited access to early rehabilitation, particularly in rural settings, may further exacerbate long-term functional limitations and restrictions in social participation.

### Strength and limitations

As a key strength of this study is its multicenter design, which enhances the representativeness of the findings. Additional strengths include the use of validated disability assessment tool (WHODAS 2.0) and the focus on an understudied population of stroke survivors in Ethiopia. However, the cross-sectional design limits the causal inference. Recall bias and residual confounding cannot be excluded. Selection bias may have occurred, as the study included only hospital-attending stroke survivors, potentially excluding individuals who were unable to access healthcare services.

## Conclusion and recommendations

A high burden of moderate to severe disability was reported among stroke survivors in Ethiopia. Older adults, individuals residing in rural areas, those in the early phase of recovery, those with comorbid conditions, those who rely on assistive devices for mobility, and those with prolonged hospitalization were at greater risk of severe disability and require particular attention. These findings emphasize the need for disability-inclusive public health strategies, expanded rehabilitation services, and equitable access to care, particularly in rural and resource-constrained settings. Future studies employing larger sample sizes, longitudinal designs, and community-based approaches are recommended to better understand disability trajectories and to inform targeted interventions.

## Data Availability

All data relevant to our findings are contained within the manuscript. Requests for further details on the dataset and queries concerning data sharing shall be arranged based on a reasonable request to the first and corresponding author (Getachew Azeze Eriku).

## Abbreviations

ADLs: activities of daily livings
AOR: adjusted odds ratio
DMCSH: Debre Marks comprehensive specialized hospital
DTCSH: Debre Tabor comprehensive specialized hospital
FHCSH: Felege Hiwot comprehensive specialized hospital
HADS: Hospital Anxiety and Depression scale
IADLs: instrumental activities of daily livings
OR: Odds Ratio
TGCSH: Tibebe Ghion comprehensive specialized hospital
UOGCSH: University of Gondar Comprehensive Specialized Hospital
USA: United States of America
UK: United Kingdom
WHO: World Health Organization
WHODAS 2.0: World Health Organization Disability Assessment Schedule 2.0
